# An Irish Radium Institute: the Royal Dublin Society and the promotion of radioactive therapy in twentieth-century Ireland

**DOI:** 10.1007/s11845-025-04035-x

**Published:** 2025-09-17

**Authors:** Adrian Kirwan

**Affiliations:** Critical Skills, Maynooth University, Maynooth, Ireland

**Keywords:** Cancer, Irish Radium Institute, Radiation, Radium, Radium Institute, RDS, Royal Dublin Society, Therapy

## Abstract

Soon after its discovery in 1898, the potential physiological effects of radium, including its possible role in combating cancer, were recognised. Its therapeutic uses led to the founding of the Royal Dublin Society’s (RDS) Radium Institute in 1914, just 2 years after the establishment of the *Institut du Radium* in Paris. In tracing the history of the Institute and the use of radium therapy, this paper explores the role played by the RDS in the relationship between the emerging science surrounding radioactivity, the scientists that were promoting and developing this, and the pioneering medical professionals that sought to use this new science for the benefit of their patients in early twentieth-century Ireland. In doing so, it demonstrates that the Irish scientists and medical practitioners actively engaged with this new medical science, assimilating, contesting and reconstructing medical knowledge in a local context.

For much of its existence, the Royal Dublin Society (RDS) was an important site for the production and dissemination of scientific knowledge. Its scientific activities were often strongly connected to its mandate as an improving society particularly agricultural improvement, demonstrated by its establishment of what was to become the National Botanical Gardens (Dublin) in 1790. Thus, an understanding of Irish Science is incomplete without recognition of the centrality of the RDS. In many of its scientific endeavours, it proved adept at securing considerable government support for its scientific work and by the nineteenth century had become an important body in the distribution of public funds for the support of Irish science, in particular scientific education. [1–3]. Despite this, the increasing importance of science saw the role that the RDS had carved out challenged.

The loss of Britain’s place as the foremost industrial nation—which was obvious to many by the middle of the nineteenth century—drove significant changes to the provision of scientific and technical education throughout the United Kingdom as it sought to maintain its economic position against emerging rivals. In counterpose to British priorities, sociologist of science, Steven Yearley argues that for the members of the RDS, science was primarily a cultural commodity rather than a tool for promoting industry [4]. The new impetus for state control of scientific and technical instruction led to the founding of the Department of Science and Art in the 1850s to direct these activities. Its establishment, as noted by historian of science Richard A. Jarrell, posed a significant problem for the RDS, which was in receipt of a six-thousand pounds annual grant for the same purposes. The result was the gradually loss of its scientific and technical educational responsibilities. Jarrell describes the RDS’s response to this as ‘defensive adaption’ in which it sought to redefined its purpose and maintain its vitality as a scientific institution [5, 6]. Consequently, with the encroachment of the state into more and more aspects of its remit in scientific education, the RDS needed new outlets to maintain its place as one of Ireland’s premier scientific societies. I have argued elsewhere that the discovery of an array of new rays at the turn of the twentieth century proved central to this ‘defensive adaption’ with the RDS purchasing radium to support the research of Irish physicists. However, the burst of research and publications that this produced quickly dwindled as the science of radioactivity advanced beyond the meagre resources of Irish scientists [7]. Nevertheless, this paper will demonstrate that radium continued as a central element in the RDS’s ‘defensive adaption’ due to its medical possibilities.

Soon after its discovery in 1898, the potential physiological effects of radium were recognised. A number of historians have studied this first atomic age, demonstrating that scientists, medical practitioners and the general public were enthralled with radioactivity and the possible impact that newly discovered radioactive substances could have on the treatment of a range of illnesses, including cancer [8–13]. In Ireland, this initially led to experimentation using radium for the treatment of cancer and skin disease, and eventually to the founding of the Royal Dublin Society’s (RDS) Radium Institute in 1914, just 2 years after the establishment of the *Institut du Radium* in Paris [10]. Despite this, the extant literature of this first nuclear age, as well as Irish historiography, is virtually silent on Irish encounters with radioactivity. Historian of science Jeff Hughes has argued that the history of radioactivity has been shaped by the development and importance of nuclear weapons, which lead to ‘a linear, teleological, “internalist” sequence of theoretical developments and associated “significant” experimental discoveries through which nuclear history could be given shape and meaning’ [14, 15]. Given the lack of such developments in Ireland, such teleological recounting of Ireland’s encounter with radioactivity was impossible. Consequently, the study of Irish cultural, social and scientific encounters with radioactivity can add much to both our understanding of this first nuclear age away of core scientific centres, as well the historiography of Irish science and medicine.

In tracing the history of the RDS Radium Institute, this paper argues that Radium and radium therapy provided a nexus for the Society’s philanthropic and scientific energies enabling the maintenance of its role as a scientific institution in the early twentieth century. It did so by promoting its centrality to knowledge surrounding, and the production of, radioactive therapeutics, as well as claiming an essential role as an arbiter of medical expertise in radium therapy. In addition, the paper demonstrates that the Irish scientists and medical practitioners actively engaged with this new medical science, assimilating, contesting and reconstructing medical knowledge in a local context. In consequence, this article will add to our understanding of the continued role of societies such as the RDS in the organisation of Irish science in the early twentieth century, the role that this sociability played in the production of scientific and medical knowledge and authority, the local construction of medical knowledge, and fill a lacuna in our understanding of Irish institutional responses to cancer in the first half of the twentieth century. In doing so, it will trace the role of the RDS in the relationship between the emerging science surrounding radioactivity, the scientists that were promoting and developing this, and the pioneering medical professionals that sought to use this new science for the benefit of their patients.

## Radioactivity and its biological effects

That radium had biological effects was discovered not long after the element itself. In 1900, Pierre Currie was to rest radium salts on his arm for ten hours, resulting in an open sore that took many months to heal and left a considerable scar [10, 16]. As shown by historian of Science Matthew Lavine, the idea that radioactive substances, such as radium, could provide some type of life force was a popular trope in this period. This combined with a lack of knowledge about its long-term consequences quickly seized the public imagination and sparked a radium craze [17]. While the Irish public was well aware of these developments, this craze was not confined to the general public. Irish researchers were also interested in the biological impacts of radium and investigated its possible uses in killing bacteria, as well as promoting growth and health in organisms [18].

The potential medical applications of radium encouraged an array of scientific experiments, which were readily reported on in Ireland. Researchers saw possibilities for the cure of various illnesses using radium; these included ‘tuberculosis, dipteria [sic], typhoid and other infectious and protozoan disease’ as well as ‘certain forms of neuralgic and rheumatic complaints [and] facial paralysis’ (Freeman’s Journal, 18 Feb. 1904). Dr Gerald Molloy—Professor of Natural Philosophy at the Catholic University of Ireland prior to his appointment as Vice-Chancellor of the Royal University of Ireland in 1903—noted the potential medical uses to which radium could be put. Highlighting that well-diluted radium bromide had been used in France to treat ‘nervous diseases’, he emphasised that such a moderately priced radioactive substance might be utilised for radioactive therapy. While discussing these possibilities, Molloy was quick to stress his lack of medical expertise. This highlights a central theme in the emergence of radioactive medicine, the need for interdisciplinarity between scientists and medical practitioners to enable the successful and safe use of radium (Irish Examiner, 11 Jan. 1904), (Irish Independent, 4 Jan. 1905). A central hope in these discussions was the element’s therapeutic potential in the treatment of cancer. As early as July 1903, the *Irish Examiner* reported on the alleged cure of cancer of the lip and palate by radium rays that had occurred in Vienna (Irish Examiner, 4 July 1903). Such reports were a regular feature in Irish newspapers in the coming years, and radium was increasingly portrayed to the public as a cure for cancer (Freeman’s Journal, 1 June 1904, 19 August 1913), (Irish Independent, 4 Jan. 1905, 15 April 1905, 20 April 1905, 26 July 1906, 29 August, 19 Dec., 29 Dec. 1913, 10 Jan. 1914), (Irish Examiner, 28 April 1905, 6 Feb. 1909).

There was also a darker side to the promotion of radium as a health product. While radium held out the promise of the treatment of illness and the restoration of health, public hopes produced a series of quack cures, which claimed the ability to treat a wide range of illnesses and offer numerous health benefits. Such claims should not be dismissed as mere lies: Short-term exposure to radium increases the production of red blood-cells and gives the appearance of improved health [10]. With no understanding of the long-term consequences of radiation exposure, the curative power of radium was a common theme in this period, with the substance often referred to as ‘liquid sunshine’ which could be readily consumed [10, 19]. In a recent monograph, historian of science Maria Rentetzi has explored the marketing of radium products, demonstrating the substantial demand generated in the US by pronouncements of the curative and health benefits of radium [20]. Ireland was not immune to this public craze for all things radium. Advertisements for radioactive cures played heavily on these notions. An example of such cures was a ‘Radium Rub’ sold at Leonard’s Medical Halls throughout Dublin. The rub supposedly gave ‘immediate relief in rheumatism, lumbago, sciatica, neuralgia, toothache etc.’ and cost 1* s.* Despite these claims, most of these cures contained no radium, which given the high cost of the element is not surprising. Irish consumers were quite lucky in this regard. Such cures, containing radioactive substances, had wreaked havoc on the health of users in the US. Given that radium has a similar chemical composition to calcium, it is readily absorbed by bones; thus, internal use is extremely dangerous. Notwithstanding this, demand for radium cures was initially strong. Radioactive spas became important sites for the radioactive craze of the early twentieth century. Ireland was not immune to this, and spas, such as in Mallow, made claims that their waters contained the element. Such claims could bring significant commercial benefit to these areas (Irish Examiner, 2 September 1904). However, while quack cures did have the potential to destroy lives, in the main, while playing on the hopes of individuals, they did little harm to the Irish public, as they contained no radioactive substances. Nevertheless, separating the reality of radioactivity from the fantasy was a difficult task for the public (indeed the ‘discovery’ and subsequent refuting of the existence of N-rays points to the difficulty for scientists as well) [21, 22].

## Radium and the treatment of cancer

While humans have suffered from cancer from time immemorial, mortality from the disease was not considered a significant problem in Ireland until the latter half of the nineteenth century. Increasing life expectancy led to a rise in the number of cancer cases that Irish physicians dealt with. Indeed, mounting concern about deaths from cancer was a global phenomenon and led to the establishment of numerous institutions, which sought to treat the disease rather than just alleviate suffering. These included the German Central Committee for Cancer Research, 1900; the (UK) Imperial Cancer Research Fund, 1902; the French Association for Cancer Research, 1902; and the American Association for Cancer Research, 1906. Despite this interest, the primary therapeutic method for treating cancer at the turn of the century was still surgical intervention [23, 24].

These international developments in the treatment of cancer were mimicked in Ireland. While surgery was still the predominant form of treatment, newer technologies were also introduced, in particular x-rays. The discovery of radium added a new weapon to the arsenal of surgeons and doctors in the fight against cancer. In Dublin, an awakening to the possibilities of radium therapy was brought about not just by an awareness of international trends in cancer treatment but also by a series of local experiments with the new element. In 1904, Christopher (C.M.) O’Brien, a surgeon at the City Hospital for Diseases of the Skin and Cancer, reported on ‘radium—the latest therapeutic remedy upon which medical science has been called on to adjudicate’ [25, p. 8]. O’Brien had applied radium to the treatment of patients and found it particularly useful in areas, such as near the eye, where it was impossible to apply x-rays. He felt it unfortunate that he could only secure five milligrams of radium. While he had had some good results, he stated that for ‘radium, to be of practical use in the cure of disease or the alleviation of human suffering, must be forthcoming in much larger quantities, of a recognised standard of activity, and at a very much cheaper rate’. This mimicked international opinion concerning the need for the application of substantial amounts of radium in therapeutics. O’Brien concluded that x-rays and Finsen Light were his preferred methods of treating both lupus and rodent ulcers, a type of skin cancer [25 (pp 8–11)]. In response to O’Brien’s paper, two Dublin physicians were to voice their positive experiences with radium therapy. A Dr Kirkpatrick and Dr Walter Smith had had success treating lupus and rodent ulcers with radium [26]. It can be seen from O’Brien’s paper, and the debate that it generated, that experimentation with radium therapy was as energetic in Dublin as the rest of Europe. This discussion also suggests that medical knowledge concerning radium was not purely of an international nature. Rather knowledge of this new science was assimilated, contested, experimented with, and finally reconstructed in a local context.

Those seeking to promote radium therapy as a viable treatment faced a number of difficulties. O’Brien had noted that the press had done those advocating such therapy no favours. The sensational pronouncements of the supposed curative powers of radium meant that the public could not be anything but disappointed by the results of its use. He feared that this would bring radium treatment, which he thought was ‘full of promise’ into disfavour. The promotion of radium therapy as a legitimate medical treatment was also hindered by the fact that ‘radio-active therapeutics could only be described as bordering on the occult’ [25 (p. 8)]. The invisible nature of radiation and claims of its effects on cancer differed little from promises made by quack cures. Thus, it was difficult for the public to separate the fiction of radium therapy from the reality.

Despite disputes concerning the use of radium, it offered advantages over the other main treatments for cancer: surgery and x-rays. While surgery could eradicate the affected cells, it involved the removal of considerable tissue to ensure that all cancerous cells were evacuated. Within months of their discovery, x-rays had been successfully applied to the treatment of cancer with a lot of success. However, x-ray therapy had a number of drawbacks. Firstly, early Crookes tubes (x-ray tubes) produced radically differing amounts of x-rays, even when using the same voltage and currents. Due to this, it was impossible to standardise treatment. Thus, practitioners would have to establish the dosage required by their x-ray apparatus for individual cases, which had the potential to be quite dangerous for patients. Secondly, the x-rays produced by early x-ray machines had relatively low energy. Due to this, while useful for the treatment of skin cancer, most x-rays were dispersed before they reached deep tumours, often leading to patients suffering skin burns. Thus, for the treatment of such cancers, they offered little alternative to surgery [8]. Radium therapy, however, presented a new option in treating deep tumours. It was easier to regulate dosage, it killed cancerous cells while doing less damage to healthy ones than surgery, and it could be inserted into the body, thus reducing damage to surrounding tissue (leading its popularity in the treatment of gynaecological cancers). In addition, radium could clear up sepsis, relieve pain and was noted for its ability to stimulate the growth of healthy tissue [27].

Two central figures in the history of therapeutic radioactivity in Ireland are John Joly and Walter Clegg (W. C.) Stevenson. In 1904, Stevenson was appointed assistant surgeon and ‘X-ray officer’ at Dr Steevens’s Hospital, Dublin, where Joly was one of the hospital governors [28]. Joly took an early interest in radium and published several papers about the element on a range of subjects [29, 30]. Thus, they represented the scientific and medical expertise needed to promote this new treatment. For Joly, the extensive application of standardised treatment methods was proof as to the value of this therapy. He argued that this allowed researchers ‘to ascribe a rational scientific basis to its legitimate claims’; thus, ‘in radio-active treatment we are pursuing methods which have been already tried extensively and found to be of definite value’ [30, p. 321]. However, for others, even while using radium successfully in the treatment of disease, its proper use and effectiveness were contested.

For surgeons, such as C.M. O’Brien, knowledge of the science in 1914 had advanced little since 1904 and they were ‘hungering, as it were, not only for some exact knowledge as to the nature and causation of cancer, but mystified more than ever as to the nature of radium and its action on living tissues, animal and vegetable’ [31, pp 337–41]. In fact, he argued that things were even more complicated than 1904 as while ‘the literature of radium-therapy has since accumulated with almost lightning rapidity, still the widest diversity of opinion exists amongst *bona fide* workers of all nations not only as to the possibilities and limitations of radium therapy, but also as to its action on human tissues in health and disease’ [31, pp 337–41]. Despite these perceived difficulties, O’Brien had continued to successfully use radium in the treatment of cancer. He maintained the opinion that large doses of radium were needed. For example, in treating a rodent ulcer, he administered 35 exposures of five milligrams of radium bromide and an additional five exposures of fifty milligrams. While the use of these substantial amounts of radium continued to be considered necessary, given its scarcity, it would be impossible to treat significant numbers of patients [31].

W. C. Stevenson, with the aid of Joly, was also to experiment with the use of radium in this period. For example, as early as 1904 while both were working at Dr Steevens’s Hospital, a patient presented with a facial rodent ulcer, which, according to Joly, was proving difficult to treat. Joly and Stevenson applied 4 mg of radium bromide to the patient with successful results [32, 33]. These medical experiments continued, and increasing numbers of patients received radium therapy. To meet this demand, Stevenson began using radium emanation (radon gas) supplied by the London Radium Institute [28, (Evening Herald, 12 March 1914)]. This had been opened in 1911 to provide radium therapy to the British populace. Patients could be treated at the Institute, but it also supplied radium emanation to hospitals and general practitioners [34].

The application of radium to therapeutic treatments was not undertaken by medical practitioners in isolation. Many scientists, in particular John Joly, took an active interest in these developments. This combination of physicians and physicists was to help advance understanding of the mechanism by which radium affected cancer. Scientists understood that radium emitted *α*-, and *β*-, particles, as well as *γ-*rays, a high-energy form of electro-magnetic radiation. α-particles travel at great speeds but they are easily stopped when they encounter skin and *β*-particles, while penetrative, are not as penetrative as γ-rays. Thus, it was *γ*-rays—which were considered ‘very penetrative and powerful ionising light waves’—that were the preferred form of radiation for cancer treatment. Hence, with the correct shielding—that blocked *α*- and *β*-particles, while allowing *γ*-rays to penetrate the patient—radium could serve as a viable alternative to x-rays and surgery. The deep penetrating γ-rays would pass through the skin to the centre of the cancer; thus, reducing the risk to the surrounding healthy tissue [30, pp 323–7, 35, 36].

The realisation of the importance of *γ*-rays was to have a significant impact on radium therapy. Radium has a number of isotopes, which, by early in the twentieth century, scientists realised were transmuting. The recognised decay series ran from radium to radium emanation (radon gas), to radium A, to radium B, and on to radium G (Lead-206). Each isotope in the series is unstable, with various half-lives. For example, the half-life of radium is *c.* 1600 years. During the process of transmutation *α*- and *β*-particles as well as γ-rays can be released. The decay of radium to radium emanation (radon), releases an *α*-particle, likewise the decay of radium emanation to radium A also releases an *α*-particle. It is not until decay of radium A that *β*-particles, with relatively high penetration, are released. Likewise, it is with the decay of radium B to radium C that *γ*-rays are released. Hence, Joly felt that ‘radium itself is of little or no direct therapeutic value’, rather it was radium B that was the true basis of radium therapy. As radium A, B and C have half-lives of 3.1 min, 27 min, 2 s, and 20 min respectively, they were unsuitable for therapists. However, radium emanation (with a half-live of 3.8 days) could easily be substituted for radium while retaining the therapeutic value of the treatment [10, 30 (pp 32–8), 36].

The use of radium emanation had several advantages: Firstly, it meant that the risk of losing the precious radium was reduced. Secondly, radium could produce many tubes of emanation, increasing the number of patients that could be treated at the same time. Thirdly, the emanation’s short half-life reduced the risk of overexposure, while also providing for a more uniform dose in comparison to early x-ray apparatus. Due to these benefits, by the 1920s, radium emanation was the main substance used for radioactive therapy [10]. However, maintaining a supply of radium emanation was not without problems. In Britain, while the London Radium Institute was capable of supplying the therapeutic needs of the capital, there were several important cities with difficulties securing a ready supply of emanation (Freeman’s Journal, 16 April 1930). The demand was such that it could take weeks for a surgeon to obtain radium emanation. The price was also considered prohibitive, with a ‘moderate dose’ for 24 hours’ use costing £6. In addition, the time taken to transport emanation created difficulties: as its half-live is around 4 days, such transportation diminished its effectiveness (Freeman’s Journal, 13 March 1914), (Evening Herald, 12 March 1914). Due to these issues, radium treatment for both the poor and rich of Dublin (and indeed the whole of Ireland) would only be possible with the establishment of such an institution in the city. The RDS was well placed to do this as ‘its laboratories not only contain the necessary appliances, but can provide the scientific supervision and delicate manipulative skill which are essential.’ In addition, ‘the council numbers amongst its members medical and scientific experts both able and willing to give their best thoughts to the ends in view—the furtherance of research and the relief of suffering’ (Freeman’s Journal, 13 March 1914). Thus, radium provided an ideal opportunity for the RDS to affirm its centrality to Irish science by demonstrating the scientific expertise it contained. As such, radium therapy emerged as an important focus of the Society’s ‘defensive adaption’ providing a new and important scientific field that demonstrated the continued importance of the RDS as a scientific institution. The Society’s role in providing a venue for the collaboration of ‘medical and scientific experts’ to advance radium therapy demonstrated its continued importance within Irish scientific and medical circles, as well as to the general public.

By the beginning of the 1910s, demand for radioactive therapy was widespread. Mercy Hospital, Cork, was to receive a gift of £120 of radium in 1911. In announcing its use in the ‘treatment of cancer, lupus, and certain varieties of chronic skin diseases’, *The Cork Examiner* made clear to its readers that the hospital would provide a treatment, which was ‘extensively used at all up-to-date surgical hospitals in America and on the Continent’. This demonstrates the link between radioactive therapy and modernity; such treatment was viewed as an essential part of any modern, cutting-edge hospital (Cork Examiner, 3 March 1911). In Dublin, ongoing experimentation with the effects of radium on cancer, lupus and skin disease had proven its worth in the eyes of some important medical practitioners and scientists (Freeman’s Journal, 14 March 1914). For example, the (Dublin) City Hospital for Diseases of the Skin and Cancer treated 412 patients using Finsen Light and Radium in January 1914 (Freeman’s Journal, 23 Feb. 1914). Reports of the success of the radium therapies being undertaken at the London Radium Institute could only increase hopes that this would prove to be a cure for cancer (Evening Herald, 31 Jan. 1914). 

## Establishing an Irish Radium Institute

By 1914, the work of number of physicians had secure a steady stream of patients seeking radium treatment. However, the future of radium therapy was still insecure. The increasing popularity of the treatment was stretching the limited supply of radium available in Dublin. A possible solution mooted to address this issue was the use of radium emanation as a substitute. However, there was no source of emanation available in Ireland. While practitioners such as W. C. Stevenson had turned to the London Radium Institute to supply emanation, this was problematic due to issues with cost and supply. These difficulties were further demonstrated when, in January 1914, two Dublin hospitals approached the RDS requesting a loan of its limited supply of radium [37].^1^ The most satisfactory solution to the issue of supply was the establishment of such an institute in Dublin. Another difficulty in expanding access to radium therapy was convincing the wider medical profession was of its usefulness as an alternative to surgery. Up to this point, radium therapy had only been applied to inoperable cancer cases and lupus; other practitioners would have to be convince of its applicability in treating a wider range of aliments. Consequently, even in 1914, radium therapy was not widely available, had limited applications and was not seen as an alternative to surgery.

Early in 1914, John Joly pressed the RDS to open a radium institute. His argument was simple: While radium therapy had its detractors, there was, in his view, no denying the increasing evidence from England and Europe that it was producing results in the fight against cancer. While it was still unknown if these cures would prove to be temporary or permanent, the impact on ‘the pathological tissues under the influence of the penetrating rays from radium’ [38 (26 Feb. 1914)] was irrefutable. In addition, although radium therapy had in many cases been unsuccessful, there could be no doubt that it had saved many after they had been declared beyond surgical intervention. Finally, even in those cases where it was unable to destroy the cancer, it had had a marked effect in relieving pain (Evening Herald, 12 March 1914). The establishment of a radium institute in Dublin would ensure a steady supply of radium emanation for Irish use, as well as reduce the problems associated with long-distance transportation [32]. For the RDS, such an institute would not only appeal to the scientific interests of the RDS’s membership but also the broader philanthropic thrust of the Society. As such, the establishment and operation of an Irish radium institute was an ideal project with which to bolster the RDS’s position as an important scientific institution. A special meeting of its Science and its Industrial Applications Committee was convened on 3 February 1914 following a request from the Society’s Council that it consider ‘the desirability of the Society increasing its stock of radium, or taking steps to promote the establishment of a Radium Institute in Dublin’. The Committee ‘unanimously recommended the council to vote the sum of £1,000, for the purchase of radium in addition to the Society’s present stock.’ Reflecting the scientific and medical interests of the Committee, radium emanation would be supplied for the promotion of radioactive research, in addition to medical applications [38 (26 Feb. 1914), 39 (3 Feb. 1914)].

In promoting the creation of such an institution, a sub-committee was drawn up to raise additional funds. Its membership, including John Joly, J. A. McClelland and A.J. Dixon, had all been involved in the promotion of radioactive science and medicine in the preceding years [7]. This ‘Radium Committee’ would be tasked with designing the scheme under which the RDS’s radium would be utilised. It would be responsible for supervising the operation of the new institute and for the purchase of additional stocks of radium. Thus, these individuals would ensure that the institutional knowledge of radioactivity that the Society had built up during the previous decade would be brought to bear on the radium institute’s formation and activities [(Irish Independent, 16 Feb. 1914), 32, 39 (3 Feb. 1914, Special Meeting)].

Central to the establishment of an Irish institute was the acquisition of a sufficient supply of radium, no mean task given the limited supply and cost of the element. On 18 March 1914 the Society requested that the physicist John Alexander McClelland return its small stock of Radium—this had been lent to him in 1904 to allow him to pursue research in radioactive science [39 (7 April 1914)]. However, this would not provide sufficient quantities of emanation to meet demands for radium therapy in Dublin. Indeed, even the £1,000 voted by the RDS for the purchase of radium was deemed insufficient and a public subscription was launched [38 (5 Feb. 1914), (Freeman’s Journal, 14 March 1914), (Evening Herald, 12 March 1914), (Irish Independent, 13 March 1914)]. Central to the success of the radium institute was its promotion in terms that would appeal to the philanthropic impulses of the Society’s membership as well as the general public. This demonstrates that despite increasing state interest and support for science, organisations such as the RDS still had an important role to play in this period. Joly was quick to act, drawing up a circular letter for a general appeal. Signed by Lord Rathdonnell, President of the RDS, it announced the £1000 donation of Viscount Iveagh (who had also contributed to the establishment of the London Radium Institute). Joly estimated that half-a-gram of radium, costing £9–10,000 would be required to meet demand. He felt that the Society was well placed to sponsor this project as the public had much confidence in it. In promoting the scheme on behalf of ‘all those who suffer from those fearful maladies which radium strives to combat [it was] entitled to appeal to the public at large for support’ [32 (p. 24), (Evening Herald, 12 March 1914)].

The prohibitive cost of radium and the radium emanation supplied from London was a central plank in the RDS’s appeal. While the £6 per dose charged by the London Radium Institute placed the cost of emanation beyond the purse of most patients, the enormous price of elemental radium meant that its purchase by hospitals in Dublin was impossible. In contrast, the RDS promised that the emanation it supplied would be charged at a nominal rate (Freeman’s Journal, 13, 17 March 1914). The undertaking of a project to realise the Irish production and application of therapeutic radium emanation allowed the RDS not only to fulfil its mandate as an improving society but also to justify its existence as a self-selecting body. The association of leading medical and scientific figures that the Society facilitated was essential to the promotion of a Dublin radium institute. In turn, this provided these members with a purpose for this association and provided a rationale for the Society’s continued existence as a scientific institution.

Iveagh’s public donation prompted the generosity of others: Sir John Griffith matched Iveagh’s £1000, while lesser donations brought the fund up to nearly £3000 [(Irish Independent, 13 March 1914), 32]. The Radium Committee’s next step was the securing of a supply of the precious element. In 1914, radium was still an extremely expensive commodity. In 1904, the Austrian government, at the time the only supplier of pitchblende (this is a uranium ore from which radium is extracted), banned its export. While some industrialists turned to mesothorium as an alternative, its relatively short half-life made it unsuitable for long-term use. The Austrian restrictions led to the mining of uranium ore in a number of locations across the globe, including Cornwall, England. However, by 1910, the USA had become the main supplier of uranium to Europe but increasing demand led to rising prices: by 1914, a gram of radium cost $120,000 [10]. The element remained scarce; by 1929, Kelly’s Hospital, Baltimore, USA, had the world’s largest supply of radium, yet this was only 5 g [8]. The RDS Radium Committee requested three tenders for the supply of radium, and by April 1914, the RDS council had approved the purchase of 200 mg of radium bromide—equivalent to 110 mg of pure radium—from the Radium Chemical Co., Pittsburgh PA, USA (a subsidiary of the Standard Chemical Company) at a cost of £3000 [32, 38 (5 March, 29 April, 7 May 1914)].

With radium secured, the next challenge facing the Society was the production and storage of radium emanation. The Radium Institute was to be based in the Society’s laboratory, located at its premises in Leinster House, Dublin. This laboratory adjoined the lecture theatre (which is now the home of Dáil Éireann) and consisted of a principal room with benches and extensive fume chambers; in addition, there were two further rooms: one equipped with air pumps of various descriptions and another with ‘balances, microscopes, spectroscopes, and other optical instruments’ [40 (p. 328)]. Central to the manufacture of a mechanism to facilitate this was R. J. Moss, who was the RDS registrar and, also, chemical analyst. The radium salts were mixed with an acid to produce a solution to aid in the production of emanation. An enclosed system was developed to extract emanation from the radium. The radium was placed in flasks, and the emanation produced taken off on Tuesday and Friday mornings using a mercury pump. The new institute would benefit from an apparatus that produced liquid air and hydrogen that was donated by William Purser Geoghegan and Samuel Geoghegan for the purpose of research and lecturing. Radium emanation is a relatively heavy gas and could be purified and then liquefied using this apparatus. This liquid could then be pumped into sealed glass capillary tubes measuring 0.7 mm in diameter, which had been designed by Moss. This tube was then divided into sections of suitable length, normally about 2 cm, depending on the requirements of the surgeon. This ensured that each capillary would produce a known amount of radioactivity; this was important as too little or too much activity could hinder therapy. In turn, the capillaries would fit into a metal exploring needle for insertion into the body [30, 32, 37, 38 (Memorandum on radium, 6 Dec. 1920), 40]. The institute soon began extracting emanation from the radium returned by McClelland. On 15 May, it was reported that 20.8 mCi of emanation had been extracted from the Society’s 41.32 mg of radium chloride [38 (15 May 1914), 39 (7 April 1914)]. Thus, the knowledge of radioactive science within the RDS was central to the Institute’s success.

The Radium Committee was also busy establishing how the new Institute would operate. The Society’s supply of radium would be kept permanently in its laboratory. Radium would only be lent for direct medical application in cases of original research. Physics research utilising this radium could be carried out, preferably at the Society’s laboratory if possible. The radium emanation would be available for both therapeutic and research purposes. For medical applications, emanation would only be supplied to qualified medical personal who had gained the approval of the Radium Committee; thus, guaranteeing its role as an arbiter of medical competence when it came to radioactive therapeutics. This not only reinforced the scientific authority of the RDS but also enhanced the credibility of doctors and surgeons engaged in radium therapy. Given the prominence of quack cures available and the unrealistic expectations charlatans had raised, this allowed medical practitioners to clearly delineate what and who was a scientific radium therapy and therapist, from what and who was not. Due to the limited supply of emanation, practitioners would be required to fill out a form from which the committee could judge the urgency of the case. Once emanation was supplied, it was stipulated that it was only to be used for treatments that had been approved (Freeman’s Journal, 14 March 1914). In drawing up these recommendations, the Radium Committee ensured that it consulted medical experts in the field of radium therapy, including W. C. Stevenson, Maurice Hayes and Edward Watson, demonstrating the importance of collaboration between surgeons with practical knowledge of radium therapy and physicists with a theoretical understanding of radium and its rays [38 (3 Nov. 1914)].

Radium emanation would be supplied by the millicurie^2^; for private patients, there would be a charge of 1* s*. 6*d*. per millicurie, while non-paying hospital cases would be charged 4*d*. per millicurie [38 (3 Nov. 1914)]. On the 25 September 1914 the Society received 244.7 milligrams of radium bromide salt, containing 110.8 milligrams of pure radium, that it had ordered from the Standard (also call the Radium) Chemical Company [37]. By 1915, the institute was readily supplying radium emanation. Over a 6-month period—1 January to 30 June 1915—it supplied 2031 mCi of emanation, for which it received £67 6* s.* 8*d.* This revenue was indicative of the low charges for emanation, particularly when compared to the £6 charged by the London Radium Institute and is demonstration of the philanthropic nature of the enterprise [38 (30 July 1915)].

## Developing the ‘Dublin method’

According to the *Freeman’s Journal,* the Radium Institute’s establishment was warmly welcomed by ‘a number of prominent [Dublin] city doctors’ (Freeman’s Journal, 14 March 1914). In 1914, radium therapy still consisted primarily of external application of a single tube of radium or its emanation. However, it was here that the new Institute faced significant difficulty. Those promoting its creation had hoped to purchase a half-a-gram of radium, which would supply 10 tubes of ‘average strength’ emanation per week. This was viewed as the minimum amount necessary to meet the needs of Dublin’s hospitals (Irish Independent, 13 March 1914). However, the Society had only been able to raise £3300 from its appeal. With this, it purchased 200 mg of radium bromide, only 2/5th of what was deemed necessary [32]. Hence, despite the best efforts of Joly and the RDS, they would be unable to meet the emanation requirements of Dublin city. Up to this point, it was generally agreed by those practising radium therapy that it was impossible to treat malignant disease with less than 50 mg of radium. They were thus left with two choices: either continue to treat limited numbers of patients or attempt to utilise radium emanation in a more efficient manner.

Joly and Stevenson were to develop a new method that would exploit radium internally using much lower doses than had hitherto been presumed necessary. Joly delivered a lecture on the subject to the RDS on 24 March 1914. The standard treatment of placing large doses of radium or its emanation on the skin of the patient entailed the use of screening (consisting of some type of metal, often lead) to protect the patient. Joly argued that by reducing the radioactivity contained in the tubes, screening would not be required. To achieve the same effect, Joly proposed replacing the single strong tube with a number of weaker ones. These could be placed in ordinary exploring needles, designed to reach more deep-seated tumours, and charged to the required activity, allowing for ‘a more controllable and uniform radiation’ (Freeman’s Journal, 25 March 1914). Using these needles Stevenson was able to treat cancer from multiple directions; thus, targeting the cancerous cells while minimising damage to healthy ones. Stevenson would surround the tumour with a number of serum needles, ranging from six upwards depending on the size of the tumour, each containing four or five millicuries of emanation in glass capillaries [36].

This technique, which was dubbed the ‘Dublin method’, gained much recognition. The necessity to innovate brought about by the scarcity of radium was shown by Stevenson’s title for the article that introduced this method to the world: ‘An economical method of using radium for therapeutic purposes’ [41]. This became an important treatment method, which was developed independently in Dublin and was disseminated from there to other radium treatment centres [42–44]. Indeed, as has been demonstrated, the ‘Dublin method’ was a direct response to the lack of radium emanation being produced by the RDS. The popularity of the method can also be ascribed to the increasing awareness of the dangers of overexposure. The Dublin method reduced this risk considerably not only was there less radiation overall but the spreading of the dose around the tumour further diminished the radiation exposure of healthy cells. In Stevenson’s experience, using this method, cancers could be treated with twenty-five millicuries of emanation [36]. This not only reduced the overall use of the precious emanation but also its cost. For the treatment of non-paying hospital patients, at 4*d.* a millicurie, the average dose would cost 8* s.* 4*d.*, far cheaper than the £6 per dose that the London Radium Institute had charged.

By 1915, radium therapy was still not considered an alternative to surgery and was primarily used in the treatment of inoperable cases. Nevertheless, its usefulness in alleviating the suffering of cancer patients and its potential as a cure were becoming more widely recognised. Stevenson carried out radium therapy not only at the Dr Steevens’ Hospital but also at a number of other hospitals throughout Dublin. Although these saw radium therapy as a ‘last resort’, it was finding a place within a broader range of medical interventions available for cancer suffers. Indeed, Robert Charles Butler (R.C.B.) Maunsell, surgeon to Mercer’s Hospital, announced that he would experiment with carrying out radium treatment first and only operating, if necessary, afterwards [45, 46]. Despite the confidence of Stevenson and Maunsell, contemporary understanding of the effects of radium, and indeed of cancer itself, was limited. Some feared that the supposed regenerative power of radium would stimulate cancer and ‘lead to more rapid dissemination’ of cancerous cells. Thus, it was argued, that without a scientific understanding of the correct dosage, which would destroy cancerous cells while not affecting healthy ones, it was too soon to attempt to replace surgical intervention with radium treatment. These arguments demonstrate much about the nature of medical knowledge concerning radiation [47].

While it was easy for scientists at the RDS to regulate the amount of emanation contained in a sample and, by doing so, to establish the dosage of radioactivity that it would release, the medical application of emanation was not as straightforward. There was little known about the susceptibility of various cancers and other aliments to radiation and how high a dosage was needed to destroy them. In addition, the ability of healthy cells to absorb radioactivity and recuperate was unknown. Thus, much of this early work by radium therapists was experimental and sought to establish a methodology for the use of radium in the treatment of various ailments. This limited knowledge of radium’s impact on both malignant and healthy tissue provided much to exercise the minds of medical practitioners. For some, it presented tantalising glimpses of the possibilities radium offered, while for others, these hopes were overblown and, potentially, dangerous. Regardless of these misgivings, and a limited supply of emanation, Stevenson’s work provided valuable experience in the application of radium emanation to malignant disease and no doubt helped in further promoting the medical applications of the treatment. His interest in this is demonstrated by his detailed reporting of the results of radium therapy to the RDS, which served as an important institution not just for the production of therapeutic radioactivity but also for the collaboration between scientific and medical practitioners and the dissemination of knowledge surrounding radioactive therapy.^3^

Historian of Irish science, Nicholas Whyte has argued that ultimately failure to adapt to the formation and politics of the Irish Free State led to the eventual demise of the RDS as a scientific institution [48]. The lack of state support for its endeavours highlights the much changed atmosphere that the Radium Institute operated in throughout its existence. However, it is important to highlight that state support was declining prior to the Free State’s formation in 1922. Thus, while the loss of patronage was a feature of the RDS’s existence following Irish independence, it also points to broader changes in state interest and support of science in this period. Despite this, the Institute’s work remained important. This was demonstrated during the transfer of the RDS from its premises at Leinster House to Ballsbridge over the course of 1924–5 to facilitate use of its former premises by the new parliament. Concerns about the continued supply of emanation during this process prompted the direct intervention of William T. Cosgrave, the President of the Executive Council of the Irish Free State. He ensured that there would be no interference with the work of the Institute until radium apparatus were installed at its new premises to ensure its work continued uninterrupted. Nevertheless, the largess of the state was never to return to the RDS, with other institutions including the expanding universities and the Royal Irish Academy increasing becoming more important centres of scientific expertise. By 1930, demand for emanation had increased beyond the resources of the Radium Institute (this was despite the loan of 412 mg of radium bromide by the (UK) Medical Research Council to the newly renamed Irish Radium Institute following representations by the Irish Public Health Council [38 (6 Dec. 1920)]) and a general appeal to RDS members and the public was initiated. This generated £10,000 and the acquisition of a further 496 milligrams of radium. The main contributors demonstrate the Society’s continued reliance on prominent members who until a few years previous had been leading southern unionist [32]. The Irish Radium Institute closed in 1952, with its supply of radium transferred to the newly opened St Luke’s Hospital. While this was the desired outcome for both the RDS and the State, it signified a number of issues. Firstly, it was recognition of the advancing state of cancer treatment and the challenges this represented to relatively limited resources of the RDS; secondly, it is indicative of the slow decline of the Society as a centre of scientific research and enquiry; finally, it demonstrates increasing state interest in supporting Irish cancer treatment (from 1920 the ‘new’ Irish Radium Institute consisted of representatives from the RDS and the Irish Public Health Council [38 (6 Dec. 1920)].

## Conclusion

As discussed at the beginning of this paper, the RDS’s role as a scientific institution was significantly challenged in the latter half of the nineteenth century. The Society operated on older model of science as a gentlemanly pursuit, which saw science as a cultural commodity not an economic one. This had served it well, allowing it to become an important scientific institution, in part due to its use of patronage networks to secure substantial state funding. However, from the mid-nineteenth century onward, the RDS’s role in the practice and teaching of science had become increasingly negated. This paper argues that radium therapy proved to be a central component in its ‘defensive adaption’ [5] as it sought to maintain its role as a scientific institution, providing an ideal nexus between its scientific and improving missions. This allowed the Society to not only garner significant financial support from both its members and the public but also draw on its vast institutional expertise. This included its laboratory and laboratory workers, but also various scientific and medical professionals who were Society members. For those interested in the therapeutic application of radium, such material support and expertise were essential but there were other benefits to be gained from this collaboration.

The early history of radioactivity is full of promises: radium’s ability to produce energy from seemingly nowhere encouraged wild claims, it was promoted as liquid sunshine that would promote health and wellbeing. At the same time, its potential to destroy cancer and other aliments presented it as a cure-all; no proclamation about its possibilities were too exaggerated. Due to this, many quack cures and quacks to sell and administer them also appeared. These impacted the public’s perceptions of this science and its practical applications, making claims that medical practitioners could never match. To counterbalance this, the authority the RDS bestowed on doctors and surgeons by its endorsement of them as qualified medical practitioners granted not only prestige but also allowed them to clearly delimitate who and what was a ‘scientific’ radium therapy and therapist, and who and what was not. The RDS did not only lend legitimacy to practitioners but also acted as a node for an emerging community of practice, where, alongside the Dublin medical journals, this emerging sub-discipline could exchange knowledge and promote a professional identity. By studying these practitioners, this paper has demonstrated the existence of an active research community where international knowledge of this emerging therapy was not passively assimilated. Rather these practitioners experimented with, contested and constructed local knowledge of radium’s potential therapeutic uses.

Finally, the history of the RDS Radium Institute and its role in the promotion of radium therapy points to broader trends in Irish science. In it, we can see the gradual demise of patronage as a central plank in the securing of state funding for science, particularly as science and science education fell increasing under the remit of the state. However, the Radium Institute’s development and central role in the provision of radioactive therapeutics points to the continued importance of such societies and associational culture in Ireland in the early twentieth century, providing important public services where the state was unable to. In doing so, it highlights the body of work that remains in tracing the social and cultural history of Irish science and medicine in the twentieth century.

## Footnotes

1 The RDS had secured a small stock of radium, amounting to 60 mg of radium bromide, between November 1903 and January 1904; source: Minute, 10 Nov. 1903 (with later additions in margins), in Committee of Science and its Industrial Applications, 1889–1914, RDS/MAN/SCI; Minute, 19 Jan. 1914, in Radium Minutes, 1914–1942, RDS ARC RDS/Man/RAD.

2 A millicurie (mCi) is a unit of radioactivity equal to 10^−3^ curie or 3X^7^ becquerel. It was originally the amount of emanation in equilibrium with 1 mg of radium. The amount of emanation is fixed due to the rate of emanation production versus its decay to radium A; hence, the curie and millicurie were measures of quantity.

3 For example, see Royal Dublin Society, Report of the council for the year 1914–30, RDS, ARC/062.
